# Leprosy prevalence spatial distribution and trend in a health region in Northeast Brazil, 2008-2017: an ecological study

**DOI:** 10.1590/S2237-96222023000200021

**Published:** 2023-09-18

**Authors:** Rayanne Alves de Oliveira, Paloma Maria Pereira de Sousa, Janiel Conceição da Silva, Lívia Fernanda Siqueira Santos, Floriacy Stabnow Santos, Lívia Maia Pascoal, Ana Cristina Pereira de Jesus Costa, Leonardo Hunaldo dos Santos, Marcelino Santos

**Affiliations:** 1 Universidade Federal do Maranhão, Programa de Pós-Graduação em Saúde e Tecnologia, Imperatriz, MA, Brazil; 2 Universidade Federal do Maranhão, Graduação em Enfermagem, Imperatriz, MA, Brazil

**Keywords:** Leprosy, Prevalence, Spatial Analysis, Epidemiology, Ecological Studies, Lepra, Prevalencia, Análisis Espacial, Epidemiología, Estudios Ecológicos, Hanseníase, Prevalência, Análise Espacial, Epidemiologia, Estudo Ecológico

## Abstract

**Objective:**

to analyse the spatial distribution and trend of leprosy in municipalities of a health region in a Northeast Brazilian state.

**Methods:**

this was an ecological time-series study based on compulsory notification of leprosy cases by the municipalities covered by the Imperatriz-MA Regional Health Management Unit, between 2008 and 2017; prevalence and mean prevalence for the period were calculated; spatial analysis of the area was carried out and maps were generated using ArcGis 10.5. Prais-Winsten regression was used for trend analysis.

**Result:**

4,029 cases of the disease were identified, and average prevalence ranged from 2.0 to 11.5 cases/10,000 inhabitants-year. The overall trend was downward. Governador Edson Lobão had the highest prevalence, 11.5 cases/10,000 inhabitants, and Lajeado Novo had the lowest prevalence, 2.0 cases/10,000 inhabitants.

**Conclusion:**

spatial distribution of leprosy cases was heterogeneous in the municipalities studied and prevalence had a falling trend.

## Study contributions

**Main results:**

A total of 4,029 leprosy cases were notified. Mean prevalence varied between 2.0 and 11.5 cases/10,000 inhab. Spatial distribution of the cases was heterogeneous and there was a falling prevalence trend over the years studied.

**Implications for services:**

These findings point to the need to strengthen active tracing strategies and expand health actions and services targeting leprosy, with the aim of increasing detection and early treatment of cases.

**Perspectives:**

It is important to carry out epidemiological investigations on the spatial distribution and prevalence of leprosy in other health regions in the state, in order to identify other areas with greater vulnerability to leprosy.

## Resumen

**Objetivo:**

analisar la distribución espacial y la tendencia de la lepra en una región sanitaria de un estado del Nordeste brasileño.

**Métodos:**

estudio ecológico y de serie temporal, con notificación obligatoria de los municipios de la Unidad de Gestión Regional de Salud de Imperatriz-MA, entre 2008-2017. Se determinaron la prevalencia y la media del período y se realizó un análisis del espacio de área, los mapas se generaron en ArcGis 10.5. Para el análisis de tendencia se utilizaron regresiones de Prais-Winsten.

**Resultado:**

se identificaron 4.029 casos y las prevalencias promedio oscilaron entre 2,0 y 11,5 casos/10.000 habitantes-año, con tendencia al descenso. Governador Edson Lobão presentó la mayor prevalencia 11,5 casos/10.000 habitantes y Lajeado Novo la menor prevalencia 2,0 casos/10.000 habitantes.

**Conclusión:**

la distribución espacial de los casos de lepra fue heterogénea en los municipios y la tendencia de prevalencia decreciente.

## Resumo

**Objetivo:**

analisar a distribuição espacial e a tendência da hanseníase em municípios de uma regional de saúde de um estado no Nordeste brasileiro.

**Métodos:**

estudo ecológico e de séries temporais, sobre a notificação compulsória dos municípios integrantes da Unidade Gestora Regional de Saúde de Imperatriz, Maranhão, Brasil, entre 2008 e 2017; foram determinadas as prevalências e a média para o período; realizou-se a análise espacial de área e os mapas foram gerados pelo aplicativo ArcGis 10.5; na análise de tendência, utilizou-se a regressão de Prais-Winsten.

**Resultado:**

foram identificados 4.029 casos da doença e as médias de prevalência variaram de 2,0 a 11,5 casos/10 mil habitantes/ano, com tendência descendente; Governador Edson Lobão apresentou a maior prevalência, 11,5 casos/10 mil hab., e Lajeado Novo a menor, 2,0 casos/10 mil hab.

**Conclusão:**

a distribuição espacial dos casos de hanseníase foi heterogênea nos municípios estudados e a tendência da prevalência, decrescente.

## INTRODUCTION

Leprosy, a communicable disease caused by *Mycobacterium leprae*, is one of the 20 neglected tropical diseases (NTDs) listed by the World Health Organization (WHO).^1^ It develops in a slow and progressive manner, leading to deformities and physical disabilities when not properly treated; ^2^ ^,^ ^3^ and continues to be a serious public health problem, especially in developing countries.^1^

In 2020, the WHO registered the occurrence of 127,396 new leprosy cases worldwide. Brazil, India and Indonesia reported 94,299 cases, equivalent to 74% of new cases detected that year. In Brazil, the case detection rate showed a sharp reduction, dropping from 17.7/100,000 inhabitants in 2011 to 8.0/100,000 inhabitants in 2020.^4^ ^,^ ^5^ It is possible that the significant reduction in cases observed in 2020 is due to the COVID-19 pandemic, which affected disease surveillance and control actions due to the overloading of health systems and services; or perhaps due to people’s movements from one place to another being restricted.^6^ ^,^ ^7^

It should also be added that tracing contracts of leprosy cases is the basis of active surveillance and constitutes an important tool for early diagnosis of new cases.^3^ ^,^ ^8^ In this sense, it is relevant to use spatial analysis tools in endemic areas,^9^ especially geographic information systems (GIS), which allow identification of space-time distributions, patterns of prevalence and transmissibility of the disease.^10^

The objective of this study was to evaluate the spatial distribution and the trend of leprosy in municipalities of a health region of a state in Northeast Brazil.

## METHODS

**Figure 1:**
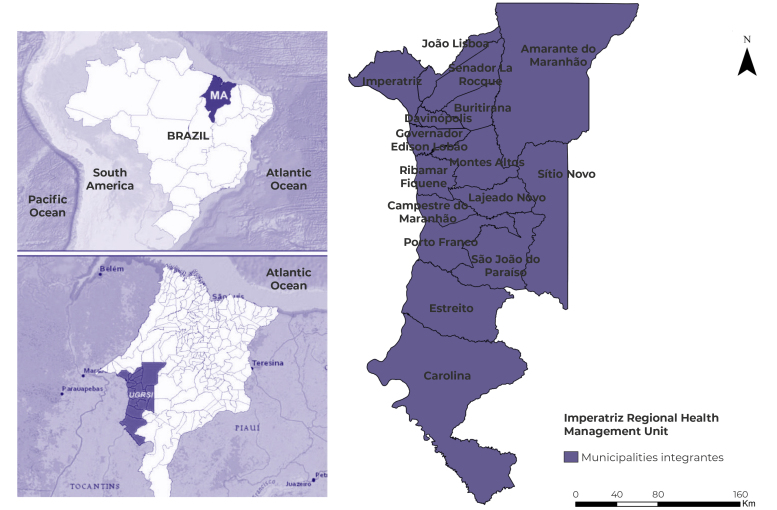
- Location of the Imperatriz Regional Health Management Unit, Maranhão, Brazil

Source: Adapted from map databases /Brazilian Institute of Geography and Statistics (IBGE).

This was an ecological time-series study, using as its ecological units of analysis the 16 municipalities covered by the Imperatriz Regional Health Management Unit (*Unidade Gestora Regional de Saúde de Imperatriz* - UGRSI), in the state of Maranhão, Brazil (Figure 1). The total estimated population within the region covered by the UGRSI is 511,735 inhabitants. The region has an area of 25,658.205 km^2^.^11^

The municipalities within the UGRSI are marked by socioeconomic inequalities and vulnerabilities: it is estimated that 24.3% of the population lives in conditions of extreme poverty.^12^ As at 2018, this health region had 128 primary healthcare centers, in addition to medium and high complexity health services available in Imperatriz, the municipality where the UGRSI has its headquarters.^13^

The study included secondary data related to the total number of leprosy case notifications held on the Notifiable Health Conditions Information System (*Sistema de Informação de Agravos de Notificação* - SINAN), for the period from January 2008 to December 2017, by municipality of residence.

Duplicated records were excluded by means of analysis using an Excel spreadsheet in alphabetical order of the full name of each case, as were diagnostic errors, based on the comparison between operational classification and the clinical forms presented. The data were obtained from the UGRSI Health Surveillance Service in September 2021.

Leprosy prevalence was calculated by municipality, dividing the total number of cases diagnosed each year by the estimated population for the municipality, multiplied by 10,000. The annual mean prevalence was calculated by dividing the total number of cases notified during the study period by 10, which corresponds to the total number of years in the series. Population data for the municipalities were retrieved from the website of the Brazilian Institute of Geography and Statistics (*Instituto Brasileiro de Geografia e Estatística* - IBGE).^14^

The analysis of the spatial distribution of the leprosy prevalence was carried out using the map database of the municipalities within the UGRSI, acquired via the Imagem/Esri company. The projection used was the Universal Transverse Mercator (UTM) with the regional geodetic system for South America - South American Datum (SAD-69).^15^ A spatial area analysis was performed,^16^ using ArcGis software version 10.5. This application makes it possible to create and manage vector and matrix data from thematic databases.^17^ The pattern of leprosy endemicity was classified according to Brazilian Ministry of Health guidelines.^18^

Prais-Winsten regression was used to analyze the prevalence trend, considering time series autocorrelation.^19^ Annual percentage change (APC) was classified as rising, stable or falling, as proposed by Antunes.^20^ For the purpose of this analysis, we used SPSS 24 (IBM SPSS Statistics, 2016). A 5% statistical significance level was set.

The research project was approved by the *Universidade Federal do Maranhão* Research Ethics Committee, as per Opinion No. 2.965.606, issued on October 17, 2018.

## RESULTS

A total of 4,082 leprosy cases were notified, of which 30 were excluded due to duplicity and 23 due to diagnostic errors, totaling 4,029 cases included in the study. 

**Figure 2:**
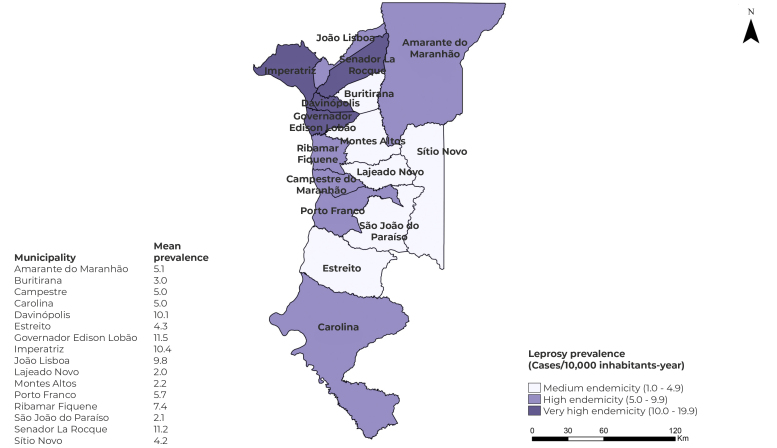
- Leprosy prevalence spatial distribution in municipalities covered by the Imperatriz Regional Health Management Unit (UGRSI), Maranhão, Brazil, 2008-2017

The spatial distribution of leprosy in the region in question was considered heterogeneous, non-random, with prevalence ranging from 2.0/10,000 inhab., in Lajeado Novo, to 11.5/10,000 inhab., in Governador Edson Lobão. The municipalities of Lajeado Novo, São João do Paraíso, Montes Altos, Buritirana, Sítio Novo and Estreito were classified as having medium endemicity, while Campestre, Carolina, Amarante do Maranhão, Porto Franco, João Lisboa and Ribamar Fiquene were classified as having high endemicity, and Davinópolis, Imperatriz, Senador La Rocque and Governador Edson Lobão were classified as having very high endemicity (Figure 2).

Table 1 shows the leprosy prevalence trend in the scenario studied. Overall, the region showed a falling trend (p-value < 0.05), with -61.1 APC over the study period. The highest overall prevalence for the Imperatriz Regional Health Management Unit was 16.2 cases/10,000 inhab. in 2011. The municipalities that showed a falling trend were Buritirana, Davinópolis, Governador Edson Lobão and Imperatriz. The trend was stable in the remaining municipalities in the period. The annual municipal prevalence ranged from 0.0/10,000 inhab., in São João do Paraíso (2008), to 23.5/10,000 inhab., in Governor Edson Lobão (2009).

**Table 1:** - Leprosy prevalence trend in municipalities covered by the Imperatriz Regional Health Management Unit (UGRSI), Maranhão, Brazil, 2008-2017

Municipalities	Leprosy prevalence (cases/10,000 inhabitants)										Annual mean^a^	Annual % change (95%CI ^b^)	p-value^c^	Trend^d^
	2008	2009	2010	2011	2012	2013	2014	2015	2016	2017				
Amarante do Maranhão	6.0	2.1	4.0	5.2	6.4	5.0	4.0	7.0	6.0	5.1	5.1	64.4 (-19.0;233.8)	0.141	Stable
Buritirana	4.0	4.0	3.0	4.0	3.0	4.0	1.3	3.3	1.0	2.0	3.0	-48.2 (-58.1;-36.0)	< 0.001	Falling
Campestre	7.1	2.4	3.0	6.0	8.1	5.1	7.2	5.0	1.4	4.7	5.0	-31.0 (-86.0;240.4)	0.593	Stable
Carolina	8.0	4.0	10.0	7.0	10.0	3.3	0.4	0.4	2.0	4.9	5.0	-81.3 (-97.6;42.3)	0.092	Stable
Davinópolis	15.0	12.4	14.0	15.0	4.0	10.5	9.0	7.0	7.0	7.1	10.1	-91.8 (-97.9;-68.7)	0.003	Falling
Estreito	6.0	6.5	7.3	2.2	2.0	5.4	4.3	3.0	3.1	3.0	4.3	-57.8 (-85.0;18.7)	0.091	Stable
Governador Edson Lobão	12.4	23.5	14.0	23.0	12.0	5.0	4.0	8.0	8.3	5.0	11.5	-97.9 (-99.9;-57.2)	0.022	Falling
Imperatriz	16.0	14.0	11.0	10.2	9.2	9.0	8.0	10.0	8.0	8.2	10.4	-83.6 (-95.2;-44.6)	0.013	Falling
João Lisboa	9.0	11.0	10.0	19.0	16.0	7.2	7.3	7.0	7.0	5.0	9.8	-79.4 (-100.0;842.2)	0.261	Stable
Lajeado Novo	1.5	3.0	1.4	1.4	4.2	-	-	4.1	1.3	3.0	2.0	3.8 (-49.2;111.7)	0.912	Stable
Montes Altos	1.1	2.2	4.2	2.1	3.2	1.1	3.3	1.1	1.1	2.2	2.2	-21.7 (-54.6;35.1)	0.323	Stable
Porto Franco	8.3	9.0	4.0	6.4	11.0	1.3	3.0	4.3	6.0	4.2	5.7	-65.6 (-92.1;50.6)	0.132	Stable
Ribamar Fiquene	9.5	8.0	11.0	17.0	8.1	3.0	1.3	9.2	5.2	1.3	7.4	-88.3 (-99.3;108.0)	0.123	Stable
São João do Paraiso	-	1.0	2.0	0.1	10.1	2.0	3.0	1.0	2.0	-	2.1	4.2 (-81.9;98.6)	0.961	Stable
Senador La Rocque	10.0	10.0	9.4	12.0	12.0	11.2	14.1	10.0	15.0	8.0	11.2	77.8 (-15.2;272.9)	0.553	Stable
Sítio Novo	2.0	1.0	1.2	22.0	2.4	1.1	5.0	2.3	2.0	3.4	4.2	-36.8 (-98.5;594.5)	0.832	Stable
Overall	7.2	7.1	7.0	16.2	8.0	5.0	5.0	5.1	5.0	4.1	7.8	-61.1 (-95.5;134.3)	0.041	Falling

a) Coefficient of the mean annual prevalence calculation; b) 95%CI: 95% confidence interval; c) Significance of the association of the Prais-Winsten regression coefficients (p-value < 0.05); d) Falling trend when p-value < 0.05 and stable when p-value ≥ 0.05.

## DISCUSSION

Leprosy showed heterogeneous spatial distribution and a falling prevalence trend in the region we studied. This points, initially, to the fact that leprosy is a neglected disease, and that in this region, which is marked by social inequalities, there are barriers to access to health services, adequate follow-up, early diagnosis and treatment.^6^ ^,^ ^12^ Generally speaking, health systems have failed to cope with leprosy due to using a disease-centered care model, without considering the social conditions in which people find themselves.^21^

Based on the spatial distribution of leprosy prevalence in the municipalities within the UGRSI, the highest prevalence were found to have occurred in Governador Edson Lobão, Senador La Rocque, Imperatriz and Davinópolis, these being places where endemicity was classified as being very high, and Amarante, Carolina and Ribamar Fiquene were classified as having high endemicity. The economic and social vulnerability present in these municipalities^21^ contributes to this finding. These are municipalities that need special attention from the health sectors, aimed at expanding disease control and surveillance actions.

Imperatriz, where the UGRSI headquarters is located, is among the municipalities with very high endemicity. An investigation carried out regarding the same ten-year period indicated out that 2,468 leprosy cases were recorded in Imperatriz, and that the spatio-temporal risk areas were associated with high population density and intense social disparities.^22^

The municipality of Buritirana was considered to have medium endemicity, and it should be noted that it borders cities municipalities such as Davinópolis and Senador Larroque, both of which have high leprosy endemicity. It is possible that cases of endemicity considerably lower than those of adjacent municipalities, as in this case, occur due to underreporting and low diagnostic capacity, whereby some residents travel to find health care in nearby municipalities. 

The overall prevalence showed a falling trend, which may be indicative of a reduction in leprosy in Maranhão. However, this may also be related to case underreporting. An ecological study identified that in Brazil and in the state of Maranhão in particular, between 2007 and 2015, there were 33,252 and 3,660 underreported leprosy cases, respectively.^23^

Another study also conducted in the state of Maranhão, between 2002 and 2011, showed that leprosy prevalence fell in the first eight years analyzed, but then rose until the end of the period. Despite the initial fall, this indicator was considered to be above acceptable, revealing health service strategy weaknesses regarding implementation of a more efficient control and prevention model.^24^

With regard to our study, despite not having investigated the programmatic performance indicators of leprosy in the municipalities within the UGRSI which had a falling prevalence trend, and also considering the incipience of studies of this nature carried out in these scenarios, it is assumed that those municipalities have in common significant progress arising from early diagnosis, increased adherence to treatment and expansion of health education actions. On the other hand, municipalities with a stable prevalence trend indicate that leprosy continues to be endemic and reveal possibly weakened health systems and services, with regard to leprosy control actions targeting vulnerable populations.^12^

As for the leprosy endemicity pattern, the geographic areas within the UGRSI considered to be of very high endemicity, with emphasis on the municipalities of Governador Edson Lobão, Senador La Rocque, Imperatriz and Davinópolis, have areas that are devoid and/or deficient in terms of basic sanitation and proper waste disposal, as well as environments with high demographic density, which contribute to the maintenance of the pathogen transmission chain and are positively correlated with higher leprosy prevalence.^25^ ^,^ ^26^

Based on data from the coverage report on Primary Care and the Family Health Strategy in the state of Maranhão,^27^ we found that higher leprosy prevalence were detected in municipalities with lower Family Health Strategy coverage. As such, Primary Health Care needs to be strengthened through training health professionals, so as to favor early diagnosis and reduce leprosy transmission.

The limitations of this study consisted of the use of secondary data, and there may be underreporting of leprosy cases. As such, we consider that leprosy prevalence we found may be underestimated.

The conclusion reached is that the spatial distribution of leprosy was heterogeneous, and its prevalence trend was falling. Thus, the need emerges to plan and implement health actions targeting vulnerable populations, such as tracing and early detection of new cases, active searching for health care dropout cases and intensification of health education, aiming at effective adherence to treatment.
